# The role of liver transplantation in the care of primary hepatic vascular tumours in children

**DOI:** 10.3389/fonc.2022.1026232

**Published:** 2022-11-24

**Authors:** Chiara Grimaldi, Jean de Ville de Goyet, Kejd Bici, Maria Chiara Cianci, Francesco Callea, Antonino Morabito

**Affiliations:** ^1^ Department of Pediatric Surgery, Meyer Children’s Hospital, University of Florence, Florence, Italy; ^2^ Department of Pediatrics, IRCCS-Istituto Mediterraneo per i Trapianti e Terapie ad altra specializzazione (ISMETT) (Institute for Scientific-Based Care and Research-Mediterranean Institute for Transplantation and Advanced Specialized Therapies), Palermo, Italy; ^3^ Department of Neuroscience, Psychology, Drug Research and Child Health (NEUROFARBA), University of Florence, Florence, Italy; ^4^ Department of Histopathology, Bugando Medical Centre, Catholic University of Healthy Allied Sciences, Mwanza, Tanzania

**Keywords:** liver vascular tumours, liver transplantation, children, hemangioma, angiosarcoma, epithelioid hemangioendothelioma

## Abstract

Liver transplantation (LT) is the standard of care for many liver conditions, such as end-stage liver diseases, inherited metabolic disorders, and primary liver malignancies. In the latter group, indications of LT for hepatoblastoma and hepatocellular carcinoma evolved and are currently available for many non-resectable cases. However, selection criteria apply, as the absence of active metastases. Evidence of good long-term outcomes has validated the LT approach for managing these malignancies in the context of specialist and multidisciplinary approach. Nevertheless, LT’s role in treating primary vascular tumours of the liver in children, both benign and malignant, remains somewhat controversial. The rarity of the different diseases and the heterogeneity of pathological definitions contribute to the controversy and make evaluating the benefit/risk ratio and outcomes quite difficult. In this narrative review, we give an overview of primary vascular tumours of the liver in children, the possible indications and the outcomes of LT.

## Introduction

Primary liver tumours represent 0,5% to 2% of all solid neoplasms in children (1). The optimal management requires a multimodal approach with chemotherapy, interventional radiology and surgery, including liver transplantation (LT) in selected cases. Two-thirds of liver tumours in children are malignant, with hepatoblastoma (HB) being the most frequent (50-60%), followed by hepatocellular carcinoma (HCC) (25-30%) and undifferentiated sarcoma (5-7%) (2). All the others, including malignant vascular tumours, are extremely rare and account for about 3-5% of all pediatric liver malignancies (1, 3). On the other hand, benign primary liver tumours such as vascular tumours, hamartoma, adenoma and focal nodular hyperplasia, represent less than 30% of primary hepatic tumours in children (1, 4).

With the implementation of new chemotherapy regimens and the development of a multi-specialist approach in recent decades (multimodal sequential or combined approach with chemotherapy, interventional radiology and surgery, including liver transplantation in selected cases), impressive progress has been achieved. In contrast, the role of liver transplantation has become pivotal for offering a chance of resection in selected patients with otherwise unresectable tumours (1, 3).

While LT for HB had been performed in children as early as in the sixties, the outcome remained poor until LT was combined with efficient chemotherapy (5). A second pivotal element was the analysis of the world experience by Otte et al., which provided evidence that primary transplantation of HB after chemotherapy was associated with excellent outcomes, while rescue LT was much less successful (6).

LT for HB expanded rapidly after, becoming the standard of care for non-metastatic, non-resectable HB. Nowadays, indications to LT are essential parts of different oncological protocols. With the greater use of LT for the treatment of HB, survival increased in a few decades, from 35% to over 90% for patients with standard-risk tumours, and 45-80% for patients with metastatic disease (7–9).

The success of introducing LT in the management of HB, stimulated its use for non-metastatic, unresectable HCC. The European Liver Transplant Registry (ELTR) overall 5-year patient (57.6%) and graft survival rates (56.3%) of children transplanted for HCC result similar to those reported by the United States Organ Sharing (UNOS) registry (5-year patient survival: 53.5%, 5-year graft survival: 42.8%) (10–12).

While indications for pediatric LT are pretty well defined for HB and HCC, the debate is still open concerning LT’s role in treating other liver neoplasms, like vascular tumours (13).

Vascular tumours of the liver are a heterogeneous group of lesions that can affect children, whose management depends on the nature (benign or malignant) and severity of clinical characteristics and presentation. Though some vascular tumours may be observed, others may necessitate an invasive, aggressive treatment (i.e. embolisation, extended resections or LT) if they present with a hemodynamic involvement (14–19).

As a matter of fact, in the setting of pediatric vascular tumours, the indications and outcomes of LT are challenging to define due to multiple factors, and the limits for concluding the existing literature can be summarised as follows: 1- first of all, the absence of large cohorts of patients even from international LT Registries as a consequence of the rarity of the diseases; 2- the heterogeneity of the terminology used (several classifications of vascular lesions, both benign and malignant) that is potentially misleading and does not allow comparing series; 3- the heterogeneity of management protocols between LT centres worldwide; 4- significant changes in treatment strategy in the last decade, with the introduction of propranolol and the subsequent difficulty to compare recent series with historical cohorts of patients. In that setting, anything better than a simple review of literature has been demanding.

According to the evolving knowledge of biology, molecular genetics and immunohistochemistry, the pathological classification of vascular lesions in the liver changed over the years. Reliability in the classification of these lesions started in the late 90s, after the separation of vascular tumours from malformations (20) and culminated in the International Society for the Study of Vascular Anomalies (ISSVA) classification (21).

The ISSVA proposed a new classification that could improve the histological definition of these lesions and clarify the different nosological entities. According to this classification, the term “hemangioendothelioma” should not be used to describe a benign lesion, but its use should be limited to “epithelioid hemangioendothelioma”, a malignant tumour (21, 22).

Historically, hepatic vascular lesions were defined according to several classifications. Dehner & Ishak (23) described 2 types of hemangioendothelioma: type 1 lesions that behave as angiomas (benign) and type 2 that indicate a borderline or low-grade malignant lesion (24). In 2021, Cordier et al. (25) added a third type of hemangioendothelioma, type 3, in which clear-cut malignant foci develop in an otherwise benign lesion. In principle, this distinction suffers from an intrinsic limitation, i.e. the sampling error due to the possibility of the co-existence of type 1 and type 2 features in a given tumour (14).

Despite the conflicting impact, the old classification has the value of underlining the concept of the potential malignant transformation of initially benign vascular liver tumours since hepatic angiosarcoma developing in infantile hemangioendotheliomas has been reported (26).

Besides that, the ISVVA classification has the merit of attempting a more clear-cut separation of vascular liver tumours into benign and malignant and establishing a definite distinction between benign hemangiomas into Infantile (IH) and Congenital (CH).

That is extremely important as the two entities differ in the clinical and pathological viewpoints. Infantile hemangioma (IH) appears after birth and grows rapidly to disappear in about one year finally. The lesion is highly responsive to propranolol, a beta-blocker, and endothelial tumour cells express the Glucose-Transporter Protein-1 (Glut-1). The congenital hemangioma (CH) grows in the uterus, is present at birth, and may have a rapid involution (Rapidly Involuting Congenital Hemangioma – RICH -), a partial involution (Partially Involuting Congenital Hemangioma – PICH -) or no involution (Non Involuting Congenital Hemangioma – NICH -). Finally, the CH is Glut-1 negative and does not respond to propranolol.

The only entity remaining undefined is the cavernous hemangioma, that represents the most frequent vascular tumour in adults (mostly an incidental finding in 20-30% of radiological series and 7% in autopsy series), while the ISSVA classification has restricted the term cavernoma to intracranial vascular malformations, and hepatologists to portal vein cavernoma which is an acquired condition.

Although a cavernous component can be observed in IH and in angiosarcoma, cavernous hemangiomas are not reported in children, the only exception being the giant hemangioma (27). The nearly exclusive ultrasound finding of cavernous hemangioma in adults and the apparent lack of a link between IH and adult hemangioma would suggest a different natural history of early infancy and adult hemangiomas (28). In contrast to the ISSVA conclusion, however, several evidences exclude a malformative nature and favour an acquired aetiology for at least some cavernous hemangiomas of the liver (29, 30).

In 1919 Foot et al. introduced the ‘hemangio-endothelium’ of the liver (31). Liver tumours of endothelial origin have been called hemangioendothelioma, further divided into two types (hemangioendothelioma type I and II) or three types, most probably caused by endothelial capillary cell proliferation, as opposed to the quiescent cavernous angioma, that represents the prototype vascular tumour from the first classification by Rudolph Virchow (23, 32).

In the attempt to obtain homogeneous clusters of patients, for this review, data extracted by the search of the literature are divided into two groups, according to the nature of the lesion (see **Table 1**): 1- benign (infantile hemangioma and lesions previously known as hemangioendotelioma type I) or 2- malignant; the latter are subdivided in a- low-grade lesions previously diagnosed as hemangioendotelioma type II, b- intermediate (epithelioid hemangioendothelioma) and c- high grade: (angiosarcoma).

**Table 1:** Synopsis of different nomenclatures.

Ishak & Dehner	ISSVA	Present Review	
**Hemangioendothelioma type 1**	Infantile HH Congenital HH	Benign	HH
**Hemangioendothelioma type 2**		Malignant	Low grade HAS
**Hemangioendothelioma type 3**	HAS		High grade (HAS)
**Epitheliod Hemangioendothelioma (HEHE)**	HEHE		Intermediate grade (HEHE)

HH, Hepatic Hemangioma; HEHE, Hepatic Epitheliod Hemangioendothelioma; HAS, Hepatic angiosarcoma.

For this review, we focused on current indications and outcomes of LT for the different types of vascular lesions. The aim was to define better the role of LT for these very uncommon and potentially life-threatening tumours. We decided to report data according to the histological and clinical diagnosis used in the various manuscripts and then separate tumours into two groups: benign and malignant lesions. Given the rarity of the diseases, the small number of cases and the heterogeneity of diagnoses, we report this literature search’s results in a narrative review.

## Benign vascular tumours

Around 30% of all liver tumours in childhood are benign, and most are vascular in origin (33).

**Figure 1:**
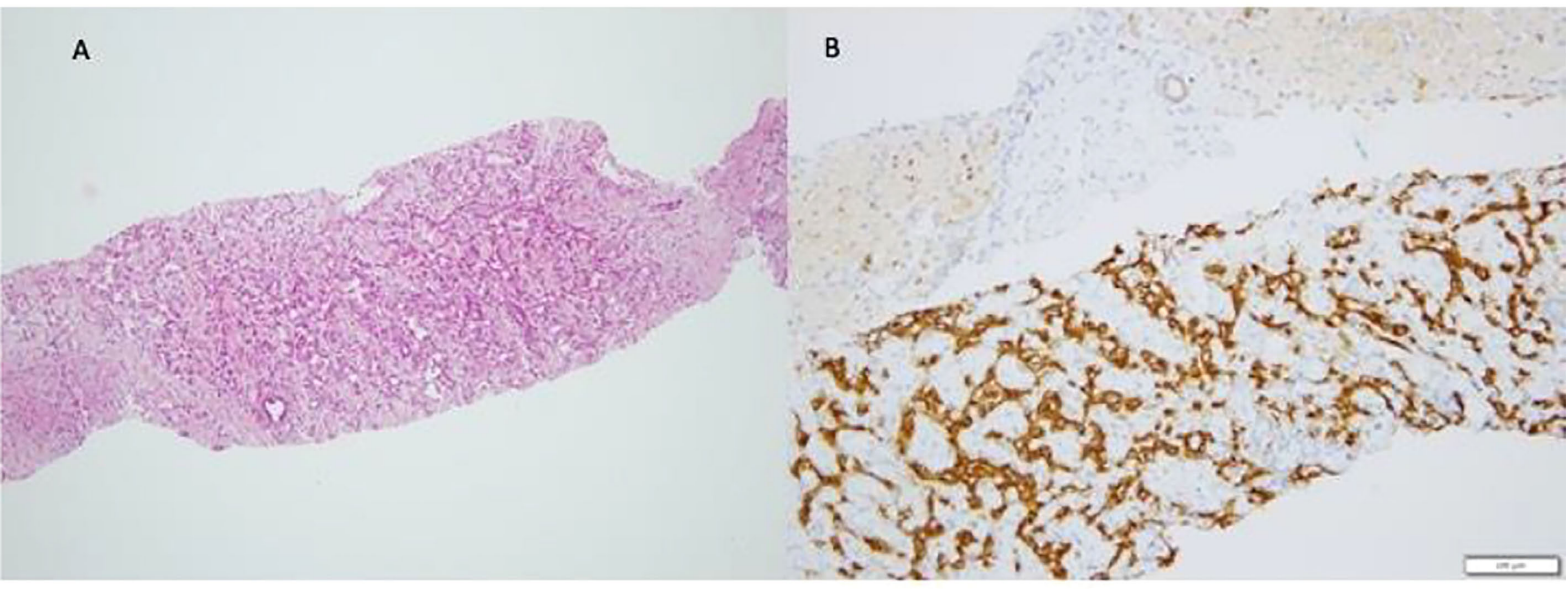
Infantile Hemangioma (IH). The typical pattern of Infantile Hemangioma is represented by proliferation of intercommunicating vascular channels **(A)** lined by a single layer ef endothelial Glut-1 positive cells **(B)**.

Hepatic hemangioma (HH) is infancy’s most common benign hepatic vascular neoplasm. Following the ISSVA classification, hepatic hemangiomas (previously known as “hemangioendotelioma type I) are now further distinguished into infantile and congenital hemangioma based on the Glut-1 staining (see **Figure 1**). To try to highlight the clinical role of LT in affected children, we will discuss all the latter (benign) lesions as a unique entities.

HH is the most common pediatric tumour, affecting 4% to 5% of infants. It exhibits rapid postnatal growth, followed by slow involution during early childhood, as early as the second year of life. The clinical presentation and associated symptoms/complications cover a wide spectrum – from asymptomatic (most of HH) to life-threatening conditions in 10% to 20%. The latter life-threatening complications are typically due to an arteriovenous-fistula effect with secondary high output cardiac failure; this can eventually lead to hepatic dysfunction, abdominal compartment syndrome (34, 35), portal hypertension with upper gastrointestinal bleeding, jaundice (36) or symptoms due to the presence of intralesional portosystemic shunts and Kasabach-Merritt syndrome (37, 38). Even if rarely described, severe hepatic dysfunction may complicate the evolution of these HH. Chen et al. reported hepatic failure as the cause of death in one patient in a cohort of 13 children treated for HH (39). Over the years, a steadily more aggressive multimodal approach has been advocated for life-threatening HH non-responding to conservative management aiming to decrease the high morbidity and mortality rates observed in these cases and propose LT in selected patients (40).

The initial management of symptomatic HH should be conservative because spontaneous regression often occurs. Historically, the initial medical intervention for HH has been corticosteroids associated with the supportive treatment of severe clinical manifestations (heart failure treatment, dialysis, ventilatory support). Regarding treatment, steroid therapy has been considered the gold standard for HH before the era of beta-blockers; however, almost 25% of the patients did not respond to steroids in a national survey in Japan (22). Other than steroids, α-interferon, chemotherapeutic agents such as vincristine, actinomycin D, cyclophosphamide, and propranolol have also been reported to be effective for the medical treatment of critical HH.

**Figure 2:**
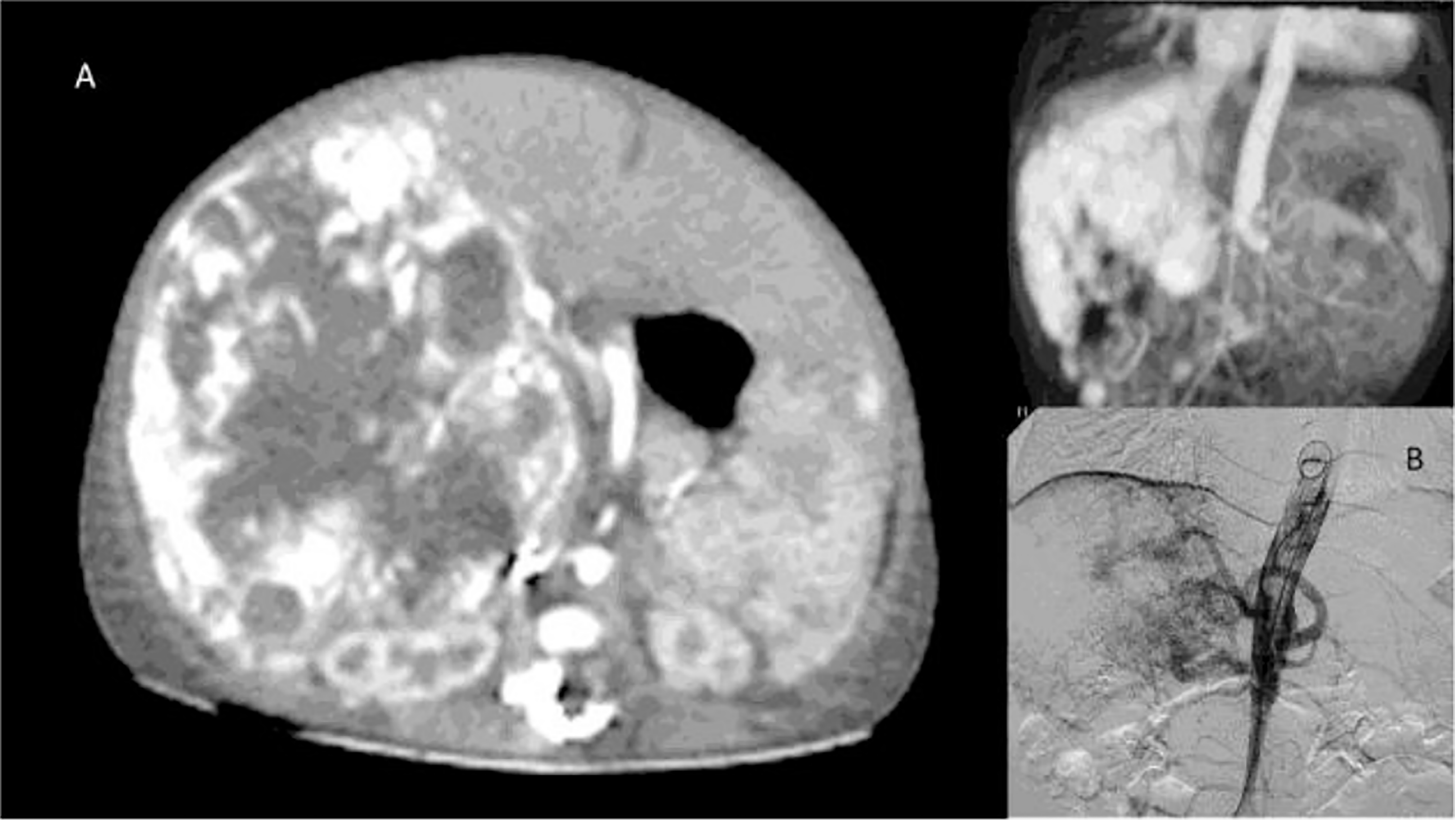
Unifocal hepatic hemangioma (HH) on CT scan **(A)** and angiography **(B)**.

Since complicated HH covers a broad spectrum of clinical manifestations, the role of surgery varies accordingly. It comes second after supportive medical management and radiological intervention in selected cases (unifocal masses, see **Figure 2**). Surgical intervention may thus be indicated in case of associated complications not amenable to stabilisation or cure by medical means. In infants who fail medical management, endovascular embolisation of the hepatic artery should be the next step to reduce intralesional arteriovenous shunting and therefore add to the medical management while waiting for spontaneous involution; this type of invasive approach has had the advantage of limiting the need to ultimately propose LT to very few cases (41, 42).

**Figure 3:**
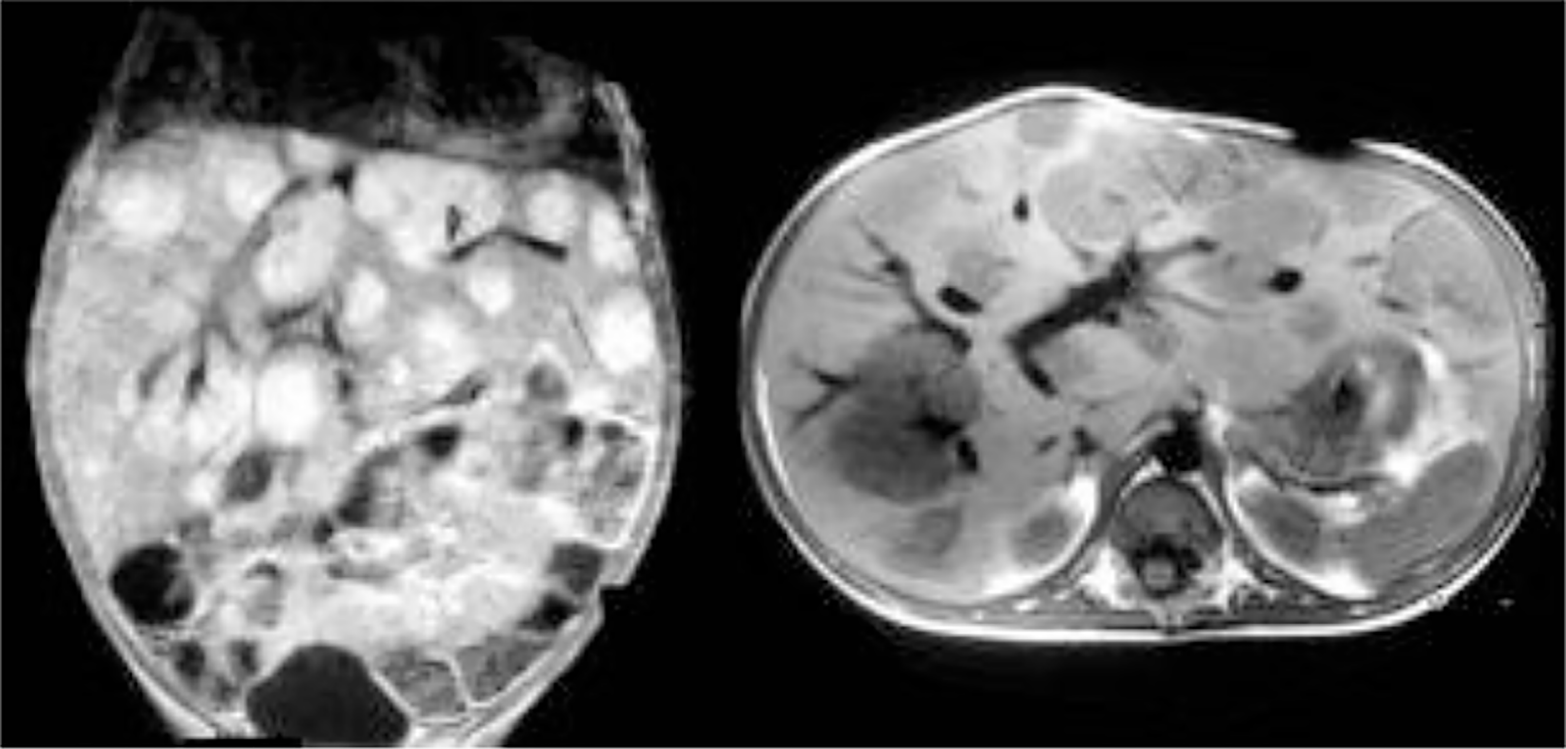
Neonatal hemangiomatosis with multifocal involvement of the liver.

As high morbidity and mortality rates (70–90%) were observed in the “non-responders”, LT has been proposed as a successful rescue strategy either in massive multifocal (also called “neonatal hemangiomatosis”, see **Figure 3**) or large unresectable unifocal lesions (14, 43).

Based on the results of the analysis of data from a Japanese national survey, Kuroda et al. developed an algorithm for management of severe clinically impaired patients. They underlined the contributing role of LT in selected children (22). The decision to transplant a child with a benign lesion may be challenging, and LT should be considered a last resource. Though the last option, LT, should be decided early enough to enable the child to face surgery in satisfactory general conditions: clinical evolution needs to be followed carefully with prompt detection of early signs of circulatory deterioration to identify those children who could benefit from LT as soon as possible (44, 45). Since hemodynamic failure may progress acutely, it may leave a very short time on the waiting list for the selection of an adequate organ donor for a usually very low-weight recipient. Despite the organ shortage for very small recipients, depending on the different transplant teams, LT has been reported to be successful both in the setting of urgent deceased donor LT and living donor LT (34, 46).

LT has been used in 2 different settings: 1- after ineffective hepatic artery embolisation and 2- in children who develop severe clinical manifestations due to intralesional portosystemic shunts.

An intrahepatic portosystemic shunt may lead to the development of pulmonary hypertension, hepatopulmonary syndrome, hepatic encephalopathy and hypoglycemia (47). Sakamoto et al. (48) reported a case of living donor LT in a child who developed severe encephalopathy and hypoglycemia due to a persistent intrahepatic portosystemic shunt after the complete involution of a HH. Optimal treatment for intrahepatic portosystemic shunts may vary and needs to be selected based on the number, size, and location of the shunts. In this specific case, the authors described ubiquitous, large and multiple shunts that led to prefer LT over multiple coil embolisation.

A review of the literature confirms the broad spectrum of presentations and complications of HH and the role that LT had taken in the management: a single centre study on the surgical management of benign lesions of the liver reported the failure of medical treatment and the need to perform LT in 4 patients out of 53 (8%): 3 of those children were affected by HH. All patients transplanted for HH were alive at the follow-up (49). Similar results were described by Kalocinski et al.: in their series, 3 infants with giant HH involving the whole liver and presenting with an associated multiorgan failure due to arteriovenous intralesional shunting were successfully transplanted after the failure of other treatments, such as steroids, cyclophosphamide, percutaneous transarterial embolisation (1 patient) and hepatic artery ligation (2 patients) (50).

Zenzen et al. (51) described an unusual case of HH in a newborn who underwent embolisation because of severe hemodynamic impairment. After the procedure, cardiac function improved, but hepatic function further deteriorated, and the patient was listed for LT, with a good long-term outcome. The authors speculated that several factors could be implicated in the failure of the embolisation procedure such as an early prenatal ischemic insult associated with postnatal factors (sepsis, total parenteral nutrition) that could be responsible for the irreversible liver damage despite the efficacy of the endovascular treatment. Several similar cases are mentioned in the literature: Markiewicz et al. (34) reported on four patients aged one to six months who underwent LT for severe heart failure and consumptive coagulopathy not responsive to endovascular embolisation due to huge arterio-venous shunting within the HH. All the patients were alive at a median follow-up of 37 months after transplantation. Also, in a series by Samuk et al. (52), two children underwent LT for life-threatening complications related to unresectable HH. Both the recipients were alive 74 and 100 months after LT, respectively.

Last: though HH-associated hypothyroidism is not an indication of LT, the latter has been shown to provide resolution of hypothyroidism, probably by means of radical resection of the liver HH (53, 54).

The last decade has seen significant strategy changes, as Propranolol was introduced for the management of complicated HH, including hepatic HH; rapidly, it was proven to be very efficient (2008), and this brought significant revisions of the therapeutic algorithm. Propranolol quickly became the first line management tool, the most effective means in controlling HH growth and decreasing the complication rate (55, 56). Since then, multiple studies have confirmed its effectiveness in patients with liver HH (57, 58).

Interestingly, Propranolol has opened a new era and modified the need for LT. Lopez-Gutierrez et al. reported that between 1995 and 2005, 7 out of 20 patients with HH were listed for urgent LT, 3 being transplanted with success, while 4 died while waiting for LT. Since 2008 however (Propranolol era), there has no need for LT and no death have been registered (59). A similar trend was observed in the UNOS database when comparing the pre-and post-Propranolol eras. Overall, the use of Propranolol has been associated with a 90% reduction of LT need and of HH-associated deaths (60).

In line with these observations, Sarıalioğlu et al. (61) also reported data on the follow-up of a group of 13 children with complicated HH managed by Propranolol in association with prednisolone. At 7-month-follow-up, 70% of patients had a complete regression of the HH. Unfortunately, no details were given about the failed cases or the use of LT.

**Figure 4:**
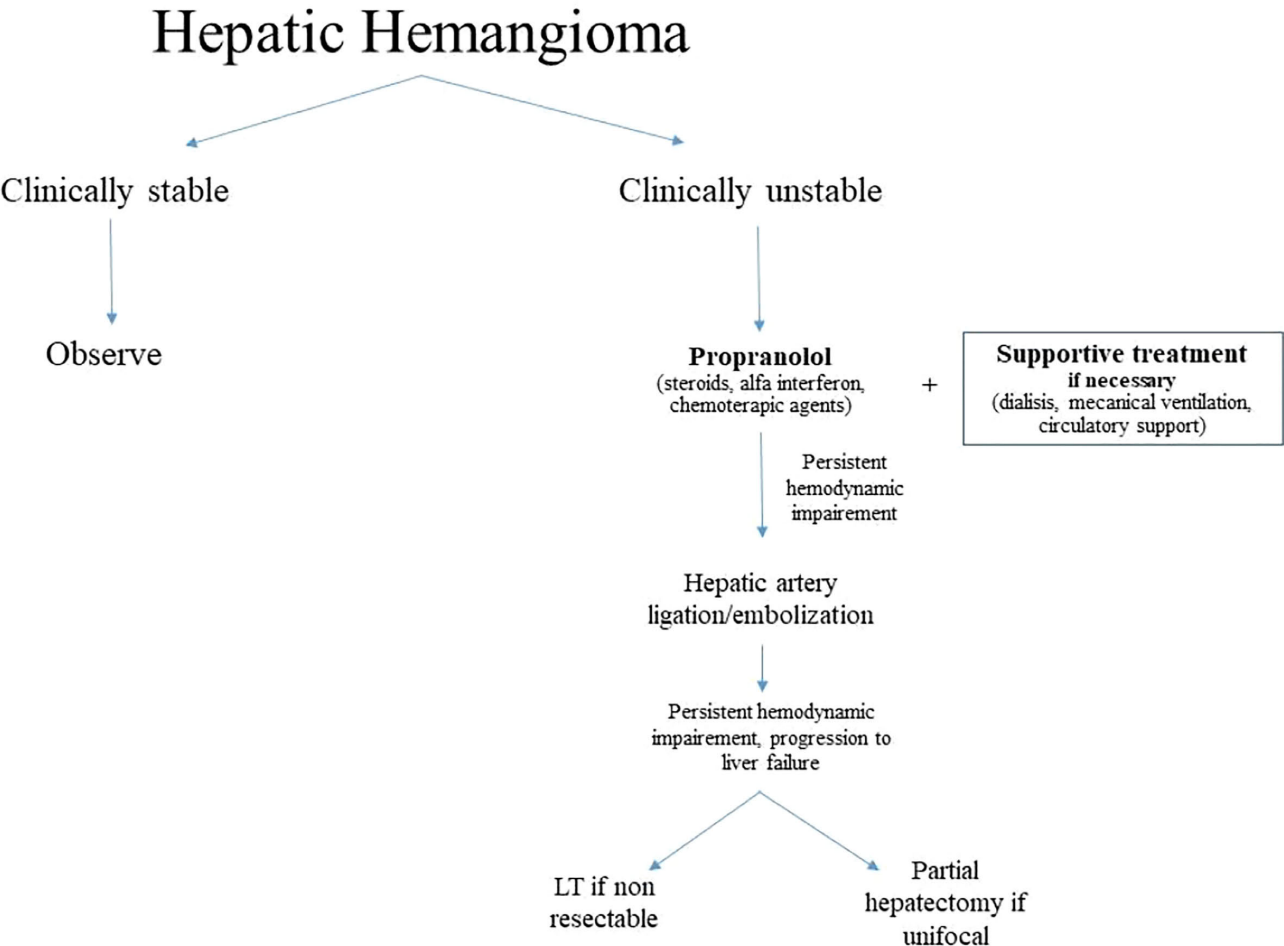
Algorithm of management for HH.

Finally, although most liver HH undergo complete resolution and disappear, some may reduce in size and leave some residue: it has been suggested that these residual masses may degenerate over the time, thus requiring attention. In other words, a prolonged follow-up could be necessary for all patients with HH that do not entirely resolve. The question of long-term follow-up in all patients remains open (24). An algorithm of management of HH is detailed in **Figure 4**, while a list of the major case-series on LT for benign vascular lesions is reported in **Table 2**.

**Table 2:** Indications and outcomes of liver transplantation and alternative treatments for children affected by benign vascular tumours of the liver reported in literature.

patients (N)		LT (N)	Indication for LT	Treatments other than LT	alive total n /LT	follow-up
Kuroda (2014)	19	1	liver failure	prednisolone(n=13), radiation therapy (n=2), embolization (n=1), liver resection (n=3)	1	not reported
Markiewicz (2009)	4	4	heart failure non responsive to embolization	steroids+vincristine (n=1), embolization(n=1), hepatic artery ligation (n=2)	4	6-36 months
Zenzen (2009)	1	1	hemodinamic and hepatic impairment	steroids+embolization	1	not reported
Samuk (2015)	3	3	cardiovascular impairment, life-threatening complications	not reported	3	74-168months
Balazs (2007)	1	1	hypothyroidism, respiratory distress, abdominal distention	steroids	1	6 months
Lee (2006)	1	1	hypothyroidism, respiratory failure	steroids+vincristine	1	7 months
Lopez-Gutierrez (2019)	31	3	not reported	propranololo (n=11)	27 (LT=3) [4 deathts while on LT waiting list]	not reported
Kulungowski (2012)	121	2	heart failure resistent to medical treatment	no treatment (n=51); steroids (n=15); steroids+vincristine/propanolol/IFN (n=17); IFN(n=2); propranololo (n=1); liver resection (n=2); unkonown (n=31)	114 [LT not reported]	not reported
Dickie (2009)	26	1	progressive caridac failure not responsive to medical therapy	no treatment (n=13); intralesion glucocorticoids injection (n=1); systemicglucocorticoids (n=7); systemic glucorticoids+vincristine (n=2); liver resection (n=1);levothyroxine (n=1);systemic glucorticoids+IFN+LT (n=1	26 [LT=1]	3-8 months

LT, liver transplantation; IFN, interferon.

## Malignant vascular tumours

Malignant vascular tumours may be separated into low and high-grade malignancies. Low grade, border-line, or intermediate malignancies comprise all cases previously classified or reported as Infantile Hemangioendothelioma type 2 and two other rare tumours: Kaposiform hemangioendothelioma and Kaposi’s sarcoma. Hemangioendothelioma type 2 is now considered a low-grade angiosarcoma.

For those who are maintaining the old terminology, the following features are recommended as diagnostic: multifocal, non-encapsulated nodules that diffusely spread through the hepatic parenchyma, vascular spaces twisting with irregular, budding, branching and anastomosing structures and papillary projections, plumps endothelial cells, multi-layered cells with hyperchromatic and pleomorphic nuclei, high rate of mitoses. In large specimens, most bile ducts are located in the central region of the nodules (62). In needle biopsy specimens, the differential diagnosis of type 1 hemangioendothelioma may be difficult because of sampling error and the possibility of the co-existence of both features (14).

The consideration of hemangioendothelioma type 2 as a low grade angiosarcoma also seems to be supported by the Zimmerman’s description of an overlapping entity between the former type 2 and a distinct form of an infantile hepatic angiosarcoma (63, 64).

## Kaposiform hemangioendothelioma (KH)

So far, no single case of primary localisation of KH has been reported. Few instances of multifocal KH in multiple visceral organs in children, including the liver, suggest that KH may have a higher potential to spread than before (65, 66). The histology looks the same in every location and consists of a combination of sheets and irregular lobules of relatively bland spindle cells defining slit-like spaces containing red blood cells, focally associated with lobules of better-differentiated capillaries and abnormal collections of small collapsed thin-walled vessels (67). Tumour cells are positive for all endothelial markers but are Glut-1 negative (68).

## Kaposi’s sarcoma (KS)

Kaposi’s sarcoma has been reported in children mostly following solid organ transplantation, including liver transplant. Tumour cells are positive for endothelial markers. In addition, they show the exclusive property of positive immunostaining for HHV-8 (Human Herpes Virus 8). The lesion usually regresses by reducing the immunosuppressant levels.

Hepatic angiosarcoma (HAS) is a high-grade malignant neoplasm and represents 1%‐2% of liver tumours in children, with around 50 pediatric cases reported in the literature (64).

**Figure 5:**
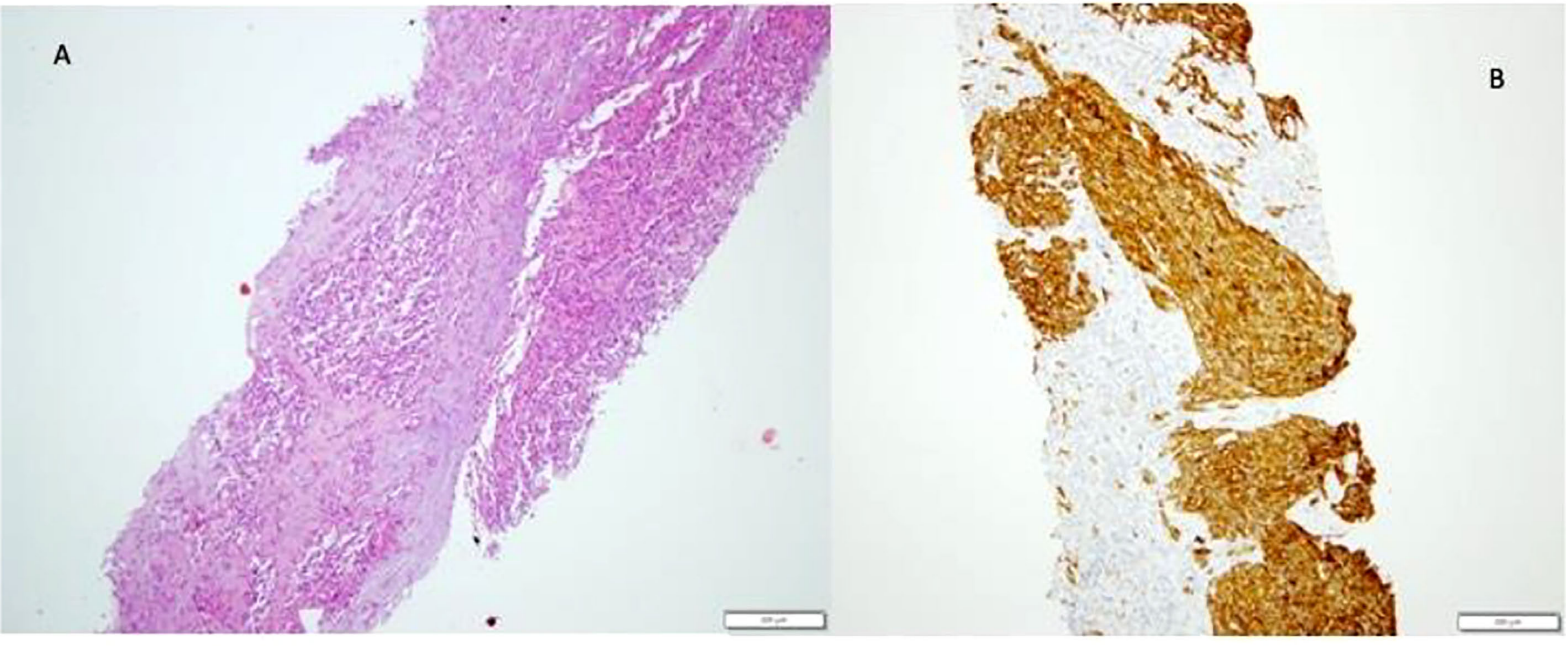
Angiosarcoma in hepatectomy specimen. Tumour mass of atypical epithelioid and spindle endothelial cells **(A)**, strongly positive on CD 34 immunostaining **(B)**.

HAS results from the proliferation of highly atypical endothelial cells with an epithelioid or spindle shape and immunohistochemical positivity to all endothelial markers (see **Figure 5**). It has been associated with different conditions, such as multiple cutaneous infantile hemangiomas (69), cutaneous mixed vascular malformations (70, 71) and dyskeratosis congenita (72). It is the most uncommon type of primary pediatric hepatic vascular tumour (73, 74), and it may arise from a hepatic infantile hemangioendothelioma which evolves towards a malignant transformation (7, 75). Recently, Sana et al. (24) retrospectively analysed a cohort of children with vascular lesions of the liver and speculated on the difficulty of differentiating malignant from benign lesions in the context of some vascular tumours for which radiological imaging can sometimes be misleading or non-conclusive. Out of 27 patients enrolled in the study, eight histological examinations were carried out, and 2 of them turned out to be HAS. These two patients had symptomatic and diffuse lesions, and both died despite treatment due to diffuse metastasis (one underwent palliative chemotherapy, and the second attempted liver transplantation). Hence, the authors underlined the importance of a correct clinical assessment and follow-up and the need to maintain a high degree of suspicion in all cases in which the clinical course of a supposed benign vascular lesion is consistently complicated because in these cases, malignancy should be suspected.

Moreover, they concluded that, although liver biopsy is at high risk of bleeding in the context of these highly vascularised lesions, it should be considered to rule out HAS in all patients older than six months of age at presentation with multifocal or diffuse suspected HH. A high degree of suspicion is recommended in all children presenting with a vascular mass of the liver after age 6 months or with a previously diagnosed HH that doesn’t completely regress and/or starts enlarging (7, 24). HAS mainly occurs in young children between the age 2 and 7 years and most frequently presents as a rapidly enlarging abdominal mass with jaundice, hepatosplenomegaly, ascites, portal hypertension, and weight loss (14, 76). Later, pain, oedema, Budd–Chiari syndrome, tumour rupture, coagulopathy and thrombocytopenia may occur (73, 77). The histological definition can be challenging because, as far as the histological findings are unique, there can be an overlap with HH, and its transformation into an aggressive malignancy may occur rapidly. At the same time, sampling biopsies can erroneously lead to diagnosing a benign lesion. Since HAS may arise on a background of a suspected benign HH, Nazir et al. (78), suggest that the risk of misdiagnosis and bleeding associated with needle biopsies may warrant the consideration of laparotomy and wedge liver biopsy. The overall prognosis of HAS is poor, regardless of treatment or stage, with a mean survival time ranging from a few months to 2 years (74). However, in 2015, Potanos et al. described a case of tumour-free survival over six years and reported a review of the literature with five cases of long-term disease‐free survival (79).

**Figure 6:**
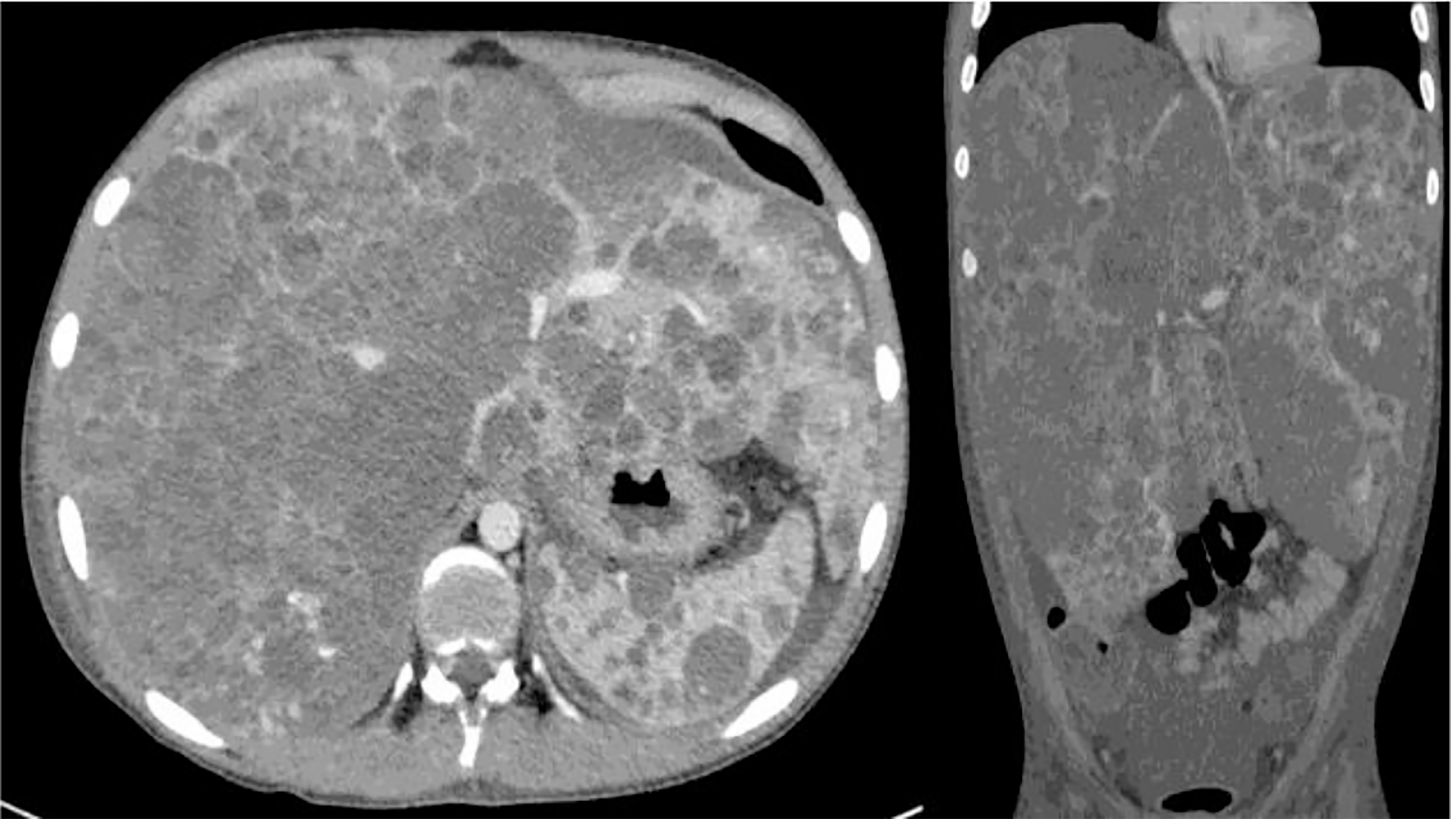
Angiosarcoma (HAS) involving the whole liver. Metastases to the spleen and intra-abdominal fluid are evident on TC scan.

The treatment of HAS is not standardised but data from the literature support the evidence that complete surgical resection is pivotal (80). Unfortunately, response to chemotherapy is scarce, and evidence of success after chemotherapy and partial hepatectomy is anecdotal (81). Moreover, HAS frequently involves the whole liver during diagnosis (see **Figure 6**), thus limiting surgical options in most cases (15, 78).

For unresectable lesions, LT could be considered. However, at the time of diagnosis, HAS is metastatic in 20-40% of children, thus contraindicating this option (73). Awan and Dimashkieh reported 4 and 1 cases of metastatic have respectively and all of them died after attempting LT (15, 17). While LT is recognised as an absolute contraindication for HAS in adults due to an unacceptable rate of recurrence (100%), the role of LT is still debated in children (82, 83). Cases of HAS recurrence following LT are also reported in the pediatric population (52). In the absence of metastasis, LT has been proposed. Pilbeam et al. (84) described a rare case of a 2‐year‐old child with localised HAS which was defined as not resectable; the patient successfully underwent an LT and received neoadjuvant and adjuvant chemotherapy. However, overall survival is less than 2 years, so transplant teams still debate whether children with non-metastatic, unresectable HAS should be listed for LT (74, 85). Data about the role of LT in children with non-metastatic HAS are inconclusive, given the deficient number of cases.

Moreover, a percentage of LT is performed on an incorrect preoperative diagnosis of HH, thus further confounding the results. Awan et al. (15) reported on three children diagnosed and treated for a clinically supposed benign hepatic hemangioendothelioma and eventually found to have angiosarcoma. The clinical course of these 3 patients was characterised by an aggressive tumour growth that did not correlate with the supposed benignity. Pire et al. (8) also experienced an unexpected diagnosis of HAS in a 3-years-old patient in the context of a previously diagnosed hemangioma which presented with rapid growth. In this case, long-term (126 months) complete remission was obtained with LT, followed by adjuvant chemotherapy. In contrast, in a large series of patients suffering from HAS who underwent LT from the database of ELTR, only 2 cases of pediatric HAS were described, but the recipients died within 2 years after LT (82). Grassia and Akerman described one case each of LT for HAS without recurrence after 5 years (64, 86).

Interestingly, Xue et al. (87) reported on an LT with an unexpected post-explant diagnosis of HAS without recurrence or metastases after 2 years. On the other hand, Sana et al. (24) illustrated contrasting results about the outcome of LT for a child with a preoperative suspected diagnosis of HH not responding to medical treatment and embolisation. Out of these two transplanted patients, one had a postoperative diagnosis of low-grade malignancy (hemangioendotelioma type 2) and was alive with complete remission at follow-up. In contrast, the other patient with a postoperative diagnosis of HAS died from disease progression 11 months after LT despite aggressive chemotherapy regimens. Similarly, in an extensive review of abdominal transplant procedures for malignancy in children, Samuk et al. (52) described a case of HAS who died 2 years after LT due to tumour recurrence in the liver graft; as a matter of fact, LT was carried out for a suspected HH, and HAS was diagnosed in the transplanted liver, strongly suggesting that it was the same tumour of the native liver.

Recently, Alden et al. (85) reviewed the literature focusing on the outcome of LT in children with HAS (16 cases, 60% survival at a mean follow-up of 2,4 years). Even if the reported follow-up is too short of drawing definitive conclusions, the authors speculate that, while adjuvant treatment alone is expected to have 25% survival at 2 years of follow-up, the association of chemotherapy and LT seems to favourably affect survival, with an increase of 2-year-survival rate up to 50%. Unfortunately, 2 of the 3 reported patients died following a relapse of the tumour, while one patient was still alive and disease-free 16 months after LT. The authors concluded that LT and adjuvant therapy currently seem to be the only therapeutic option for HAS in children.

Interestingly, the successful use of mechanistic targets of rapamycin (mTOR) Inhibitors as immunosuppressive agents post LT for HAS has been reported by Alden et al. (85) based on the presence of recurrent mutations in genes of the PI3K/mTOR and RAS/MAPK pathways and high responses to mTOR inhibition in an angiosarcoma mouse model (88).

## Hepatic epithelioid hemangioendothelioma (HEHE)

Hepatic epithelioid hemangioendothelioma (HEHE) is a rare, low-to-intermediate grade tumour of vascular origin (<1 per million children) (73, 89). It occurs mainly in adolescents with an intermediate behaviour characterised by a slow growth but associated with the risk of local recurrence and distant metastasis (13, 90–93).

Clinically, HEHE usually presents with upper abdominal or epigastric pain, weakness, jaundice, hepatosplenomegaly, weight loss and impaired general condition. Anicteric cholestasis and cytolysis are reported in 60%, and 40% of cases and vascular compression or infiltration may result in portal hypertension, Budd–Chiari-like syndrome, liver failure and pulmonary symptoms (10% of cases) (73, 90). Around 20% of patients are asymptomatic.

HEHE may present as a unifocal large liver mass or multinodular lesion extending to the whole liver (73). Multiple calcifications are reported in 20% of cases, and combined micro- and macrovascular invasion are described in half of the patients’ (7, 73).

**Figure 7:**
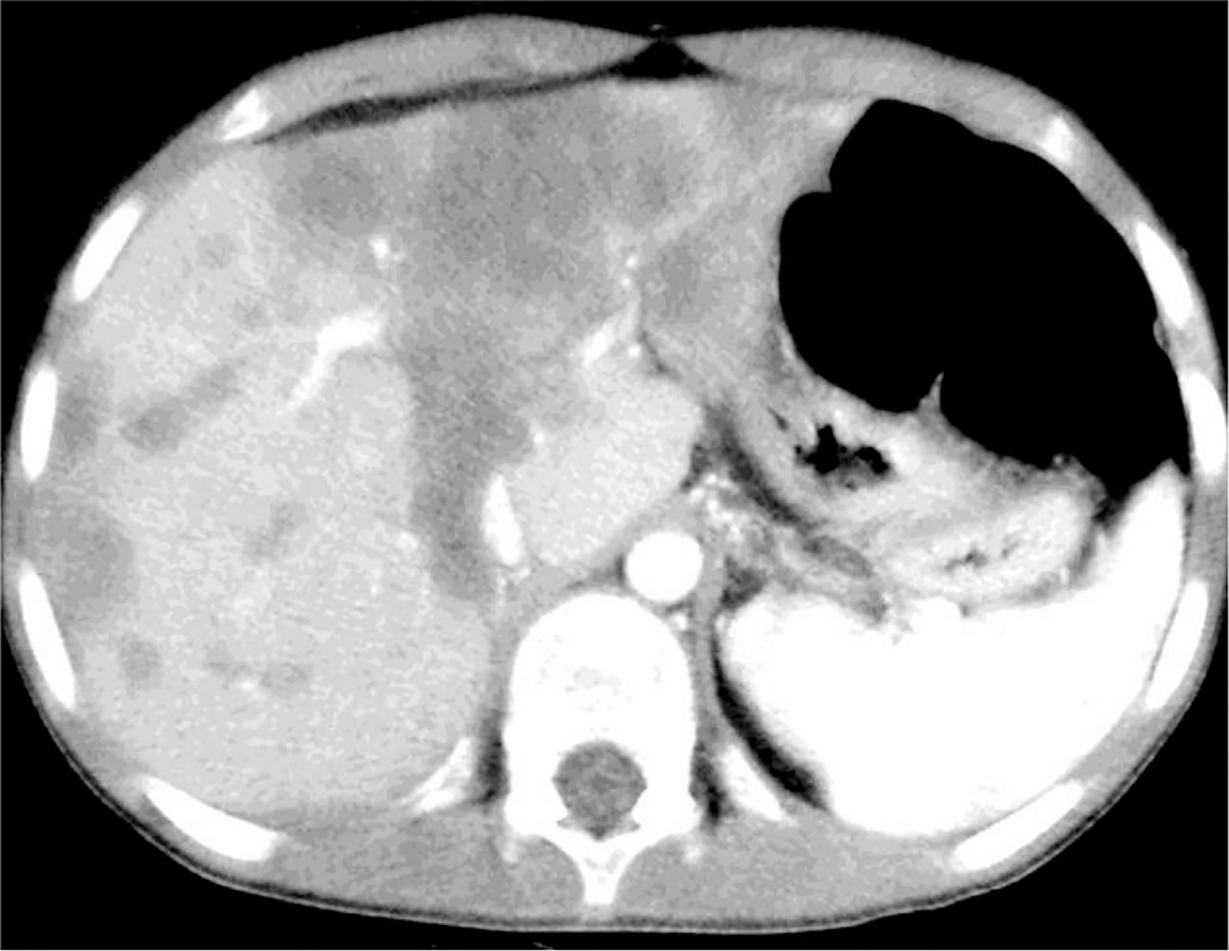
Radiological aspect of hepitalial hemangioendothelioma (HEHE) on CT scan.

**Figure 8:**
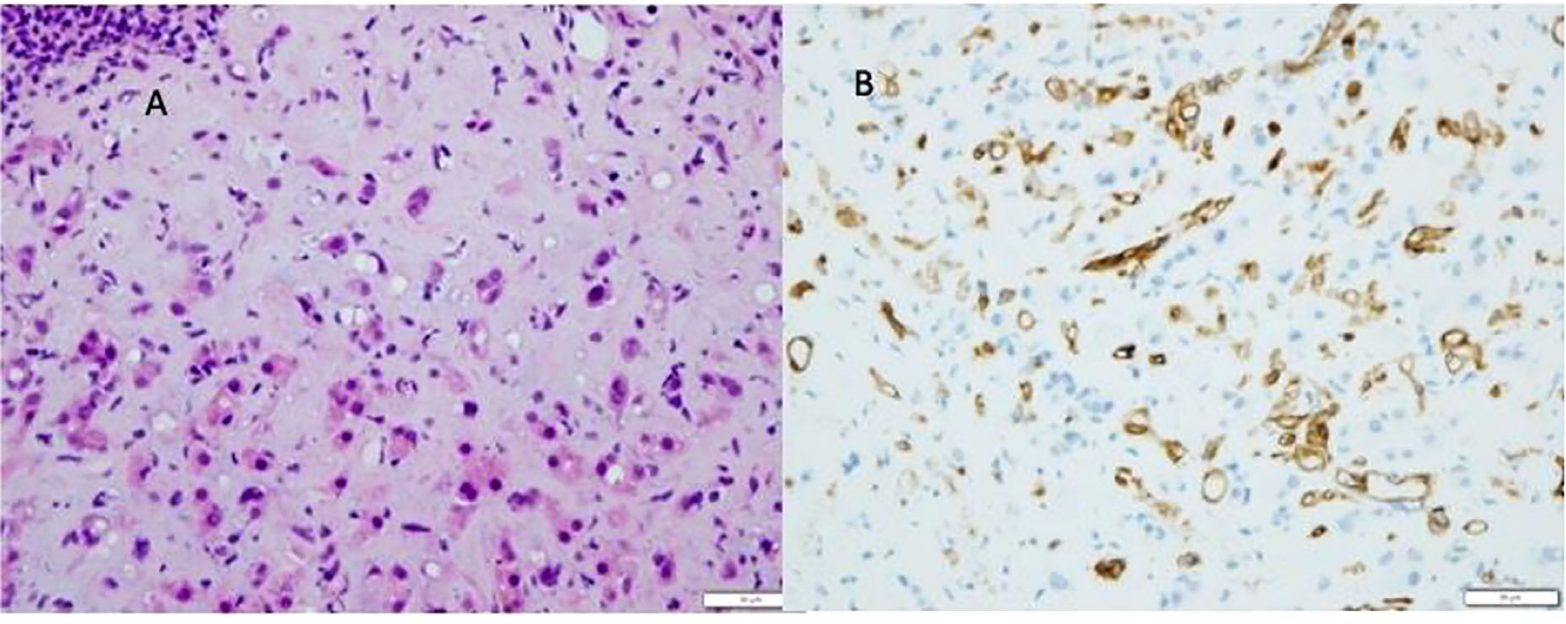
Epitheliod hemangioendothelioma (HEHE). Proliferation of atypical endothelial cells with a vacoulated appearance **(A)**, positive to CD34 immunostaining **(B)**, in a myxohyline stroma.

Although both clinical and radiological features raise suspicion of HEHE (see **Figure 7**), the pathological examination is mandatory for the definitive diagnosis (7, 73). Indeed, the histological features are highly characteristic and consist of scanty vacuolated malignant tumour cells in a myxohyaline stroma, often showing intravascular growth. The vacuolated cells are favourable to all endothelial markers (see **Figure 8**)

HEHE is extremely rare in children, and it is not definitively ascertained whether its behaviour in children is similar to that of adults (18). The high rate of multifocality associated with indolent clinical behaviour is the reason for proposing LT as the treatment of choice in adults (94–97). LT usually has good outcomes in adult recipients, even in patients with extrahepatic disease: 5-year survival following LT is up to 70% in different series Fields (73, 94, 98–101).

Overall, a few cases of LT for HEHE in children are reported, and data on outcomes of LT in this population are scant. Makhlouf et al. (102) collected data from a large cohort of 137 HEHE, but most of the patients were adults. Indeed, out of this series, only seven cases were aged less than 20 years, and, unfortunately, their management and outcome were not analysed separately. Hence no additional considerations could be evoked. Concerning the pediatric population, in a multicentre series, Sharif et al. (90) collected data from 6 children. Three of them underwent LT: indications were a liver failure in 1 case and Budd—Chiari syndrome in 2 cases, respectively. All patients died, one due to non-tumour related reasons, while 2 developed tumour recurrence in the transplanted graft and distant metastasis, dying shortly after transplantation. Interestingly, histology showed recurrence in the liver graft as angiosarcoma. The authors concluded that HEHE in children may have a more malignant behaviour than adults and that LT alone may not be an excellent therapeutic option.

Meanwhile, rare cases of successful LT for HEHE can be found in the literature. Taege et al. (103) described the clinical course of one child with a slowly progressing HEHE without the extrahepatic disease who did benefit from LT and attained a 3 years disease-free survival. Samuk et al. (52) reported one case of metastatic HEHE at diagnosis, who was treated by adjuvant chemotherapy with regression of intrahepatic disease and effective lung metastasis control. LT then followed chemotherapy with a 5-year disease-free survival after transplant.

In a fascinating analysis of data from the UNOS database, Guiteau et al. (104) reported the experience of LT for pediatric HEHE. The five-year patient survival rate was 60.6%, and the recurrence rate 2.8%. The death rate caused by recurrence was 9%. Unfortunately, the study by Guiteau et al. did not investigate the reasons for this non-favourable outcome for HEHE, despite a low recurrence rate.

Although rare, and with a general trend following transplantation that does not allineate with the good outcome of LT for adults, HEHE is still the third most common pediatric, unresectable liver tumour that may benefit from LT (18), but more evidence is required to standardise this therapeutic approach.

In addition, the relationship between HEHE and HAS is an attractive issue which should deserve attention. As previously mentioned, recurrence, as HAS in liver grafts of patients transplanted for HEHE, is reported, and foci of HAS may be detected in specimens of HEHE. Overall, data from the literature, even if scarce, suggest that outcome of HEHE in the pediatric population is less favourable than in adults.

**Figure 9:**
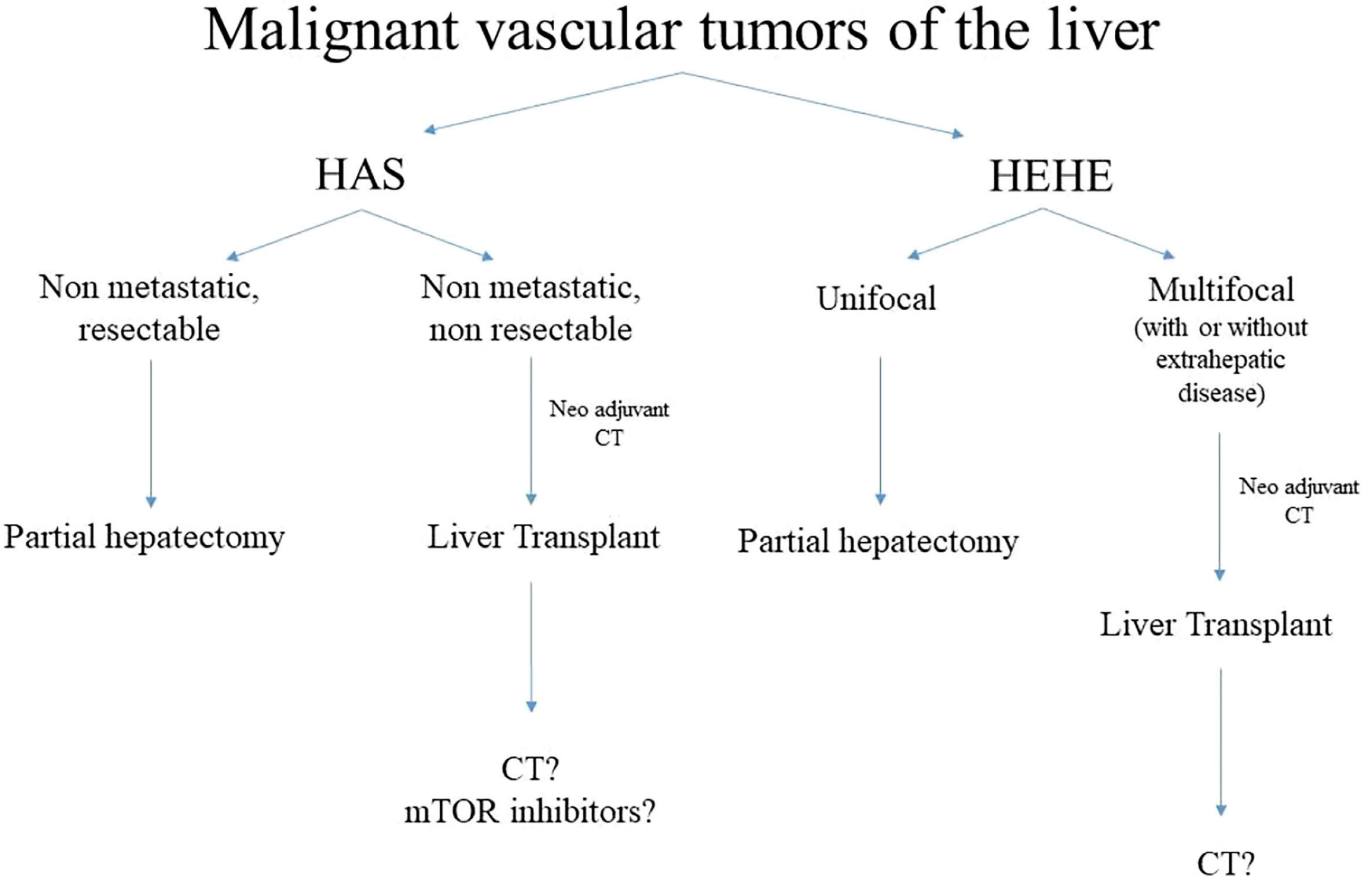
Algorithm of management of malignant vascular tumours. HAS, hepatic angiosarcoma, HEHE, hepatic epitelioid hemangioendotelioma, CT, chemotherapy.

Recently, a large study collected data from 699 patients with hepatic vascular malignancies from The National Cancer Database (105). Only 16 patients (2%) were children, six (1%) with HAS and 10 (5%) with HEHE. The median age at presentation of HAS was 7 years, while it was 15.5 years in patients with HEHE. The present study focused on the role of surgical resection rather than LT, two (33%) patients suffering from HAS and six (60%) patients with HEHE underwent surgical resection: the overall mean survival rate at 1-, 3-, and 5-year was 67%, 50%, and 50%, for those affected by HAS and 90%, 90%, and 90% for those affected by HEHE. Interestingly, in both conditions, the survival rate was higher when compared with the adult populations. Despite the lack of a systematic approach, an algorithm for management of vascular malignancies is summarised (see **Figure 9**). **Table 3** highlights the current literature on LT for malignant vascular tumours of the liver.

**Table 3:** Indications and outcomes of liver transplantation and alternative treatments for children affected by malignant vascular tumours of the liver reported in literature.

	Vascular tumor (N)	LT (N)	Treatments other than LT	Indication for LT	Alive [LT]	Follow-up
Andres (2007)	HAS (n=1)	1	steroids, interferon	hemodinamic impairment, not resectable	0	6 years
Pire (2021)	HAS (n=1)	1	liver resection	recurrence after liver resection	1	12 years
Selby (1992)	HAS (n=10)	0	liver resections + cisplatin+adriamicyn+ radiotherapy+ artery ligation (n=3),liver biopsy+actinomycin+steroids+ artery ligation (n=2), unkonwn (n=5)		1 [LT=0]	0-27 months
Awan (1996)	HAS (n=4)	1	liver resection (n=1), vincristine+cyclophosphamide+adriamycin (n=2)	unresectable	0	4 mo
Falk (1981)	HAS (n=4)	0	Radiation therapy+CT+steroids (n=2),radiation therapy (n=1), artery ligation+radiation therapy(n=1)		0	4-45 months
Dimashkieh (2004)	HAS (n=1)	1	IFN+steroids+embolization	massive bleeding	1	14 months
Alt (1985)	HAS (n=1)	0	vincristine+doxorubicin		0	not reported
Sana (2021)	HAS (n=2)	2	INF+embolization (n=1), Steroids+IFN (n=1)	failure of medical treatment and embolization	1	11-54 months
Kamath (2015)	HAS (n=1)	0	steroids+propranololo+cinblastine+methotrexate (n=1)		0	not reported
Foote (1919)	HAS (n=1)	0	laparotomy, no liver resection		0	1 day
Samuk (2015)	HAS (n=1), HEHE (n=1)	2	none	misdiagnosis of HH, uncontrolled bleeding, not resectable	1 (HEHE)	2-64 months
Grassia (2017)	HAS (n=3)	3	steroids+embolization+IFN+cisplatin+doxorubicin+radiation (n=1),ifosfamide+doxorubine+sirolimus (n=1)	not reported	2	9 months-5 years
Nakaya (2014)	KH (n=1)	0	steroids	Kasabach-Merrit syndrome, bleeding, respiratory failure	0	9 days
Nord (2006)	HAS (n=1)	1	steroids+vincristine+arterial embolization (n=1)	cardiac failure	1	12 months
Al Dhaybi (2010)	HAS (n=1)	0	steroids+embolizations+vincristine (n=1)		0	10 months
Kirchner (1981)	HAS (n=1)	0	steroids+vincristine+cytoxan+adriamycin+5-fluorouracil+radiation therapy+liver resection(n=1)		1	2 years
Weinber (1983)	HAS (n=10)	0	liver resection (n=3), radiation (n=2), steroids (n=2), radiation+CT (n=1), radiation+ arteryligation (n=1), radiation+steroids (n=1)		1	6 years
Nazir (2006)	HAS (n=2)	0	steroids+INF+CT (n=1); no treatment (n=1)		0	3 months
Potanos (2015)	HAS (n=1)	0	bevacizumab+paclitaxel+ liver resection (n=1)		1	6 years
Deyrup (2009)	HAS (n=2)	1	liver resection+CT (n=1), liver resection (n=1)	not reported	0 [LT=0]	0-11 months
Gunawardena (1997)	HAS (n=1)	0	adriamycin+cisplatin+liver resection (n=1)		1	44 months
Orlando (2013)	HAS (n=6)	6	steroid + liver resection (n=1), CT (n=2), radiotherapy and steroid-interferon therapy (n=2)	unresectable (5), intractable bleeding after liver resection (1)	0	< 2 years
Pilbeam (2018)	HAS (n=1)	1	doxorubicin+docetaxel+ifosfamide+ steroids (n=1)	unresectable	1	16 months
Alden (2021)	HAS (n=3)	3	steroids+radiation therapy+interferon+muronomab+azathioprine+cyclosporine (n=1), steroids+propranolol+tacrolimus+mycophenolate mofetil (n=1),steroids+everolimus+tramet(n=1)	unresectable	1 [LT=1]	6-22 months
Ackerman (2011)	HAS (n=5)	1	steroids+radiation therapy+embolization (n=2),steroids+ liver resection+radiation therapy (n=1), embolization+liver resection+interferon (n=1)	relapse and cardiovascular impairment after medical treatment and embolization	1 (1) [5y]	5 years
Xue (2014)	HAS (n=1)	1	sirolimus (n=1)	unresectable	1	27 months
Ferrari 2002 (86)	HAS (n=1)	1	chemotherapy	unresectable	0	15 months
Sharif (2004)	HEHE (n=5)	2	liver resection+vincristine+actinomycin+ifosfamide(n=1), actinomycin+ifosfamide (n=2)	unresectable	3 [LT=0]	2-26 months
Lerut (2007)	HEHE (n=2)	2	not reported	not reported	74% survival (adult+children)	10 years
Makhlouf (1999)	HEHE (n=7)	7	not reported	unresectable	not reported	not reported
Taege (1999)	HEHE (n=1)	1	Vincristin+adriamycin+actinomycin D+Ifosfamid+steroids (n=1)	unresectable	1	3 years
Guiteau (2010)	HEHE (n=35)	35	not reported	unresectable	21	5 years
Commander (2021)	HAS (n=6)	not reported	liver resection (HAS=2, HEHE=6)		HAS=3, HEHE= 9	5 years

LT, liver transplantation; IFN, interferon; CT, chemotherapy; HAS, hepatic angiosarcoma; HEHE, hepatic epitelioid hemangioendotelioma.

## Conclusions

LT is the treatment of choice for end-stage liver disease and provides excellent outcomes and post-transplant quality of life. Though proposing LT for the treatment of HB was slightly controversial at the beginning, the publication by Otte et al. in 2004 was pivotal for confirming the role of LT in managing unresectable HB (6).

A systematic approach that includes preoperative chemotherapy and surgery is essential to maximise long term outcomes. According to data derived from large series and international Registries, sequential use of chemotherapy and LT determines a 5-year survival rate of up to 86% for PRETEXT 4 and 3HR HB, provided lung metastases are cleared before transplant (106).

Similar indications have been applied to the management of HCC, even if outcomes are less optimistic, with a reported five years survival rate of 53.5% - 57.6% (10–12).

Both HB and HCC are rare tumours but, taken together; they account for around 90% of all primary hepatic neoplasms in children (107). Therefore, the large international LT databases allow data collection from statistically adequate cohorts of patients.

Currently, non-metastatic unresectable HB and HCC and some complicated benign liver masses are recognised as formal indications of LT, but the role of LT in treating vascular malignancies is still debated. Providing evidence by analysing data from historical series is complex and limited by the small size and heterogeneity of the published series and the variations of the used terminology. This limits an efficient data search and opportunities for analysing outcomes in the latest specific indication. To have homogeneous groups of patients, we reported the indications and outcomes of LT by separating benign from malignant lesions. Even if benign vascular tumours of the liver are frequent, they rarely need treatment and even more infrequently, they may indicate LT.

Before introducing propranolol as a treatment for HH, indications for LT were limited to severely ill patients with hemodynamic decompensation. For these highly selected patients, mortality was associated with the tumour-related preoperative severe multiorgan impairment and difficulty matching an adequate donor (low recipient weight, short waiting list time) for transplantation. Moreover, in the era of propranolol, the number of patients needing LT for this indication is further reduced, and LT for this specific indication has become so exceedingly rare that some recent series demonstrate the disappearance of these patients from the waiting lists. On the other hand, even if LT is the only possible surgical option for frequently unresectable vascular malignancies, reports on good long-term outcomes are almost anecdotal.

Over the last decades, long-term outcomes of children who undergo LT constantly improved, thanks to tailored immunosuppression, pre-emptive and aggressive follow-up and treatment of medical and surgical complications (108), and optimisation of the allocation policies for pediatric recipients. In particular, specific algorithms for organ sharing and liver splitting (109), together with the dissemination of living donor LT programs, currently grant many grafts. The established benefits of LT, associated to relatively easy access to liver grafts, allow expanding the pool of recipients with unusual indications of LT, such as rare, poor chemo-sensitive, non-resectable tumours. The subgroup of vascular malignancies could be a challenging but beneficial field of development, hence the pediatric transplant community should adopt a more aggressive approach to these patients who probably will, in the next future, benefit from a combination of LT and new emerging drugs. The introduction of new neoadjuvant drugs and more appropriate immunosuppressive regimens, such as mTOR inhibitors, seems a viable option to improve the fate of these children. However, more evidence is needed to corroborate these hypotheses, and, hopefully, a more homogeneous classification will help cluster larger groups of patients and obtain robust data.

## Author contributions

CG and KB contributed to conception and design of the study. MC organized the database. CG, KB, and MC performed the statistical analysis. CG wrote the first draft of the manuscript. All the authors wrote sections of the manuscript. All authors contributed to manuscript revision, read, and approved the submitted version.

## Conflict of interest

The authors declare that the research was conducted in the absence of any commercial or financial relationships that could be construed as a potential conflict of interest.

The reviewer AL declared a past co-authorship with the authors MC and AM to the handling editor.

## Publisher’s note

All claims expressed in this article are solely those of the authors and do not necessarily represent those of their affiliated organizations, or those of the publisher, the editors and the reviewers. Any product that may be evaluated in this article, or claim that may be made by its manufacturer, is not guaranteed or endorsed by the publisher.
